# The Story of the Insane from Year to Year

**Published:** 1897-08-14

**Authors:** 


### THE STORY OF THE INSANE FROM YEAR TO YEAR.

Tenth Series.

### THE BOROUGH ASYLUM, PLYMOUTH.

This is a small asylum, containing only 243 patients. There were 45 admissions, 26 recoveries, and 16 deaths. The recoveries, calculated on the admissions, stand at 63·41 per cent., which is the highest we remember meeting with in a public asylum; and the deaths, at 6 42 per cent. on the average number, are very low. Intemperance in drink caused the insanity in 7 of the year's admissions, and hereditary tendency in 14. Dr. Davis says he is "each year less inclined to engage nurses who have served in other asylums, as he finds their influence decidedly discouraging." We believe he is right here, and several asylums already act on the principle. It is much to be desired that every superintendent could be imbued with similar views. The committee express their high appreciation of the zeal and ability of the medical superintendent. The weekly rate of maintenance is 11s. 2½d. per head per week.

